# The effect of hospital-based violence intervention programs on post-traumatic stress disorder symptoms among violent assault victims: a systematic review

**DOI:** 10.1186/s40621-026-00694-1

**Published:** 2026-06-10

**Authors:** Clay Schuler, Margaret McAlister, Joshua Ellis, Nakita Lovelady

**Affiliations:** 1 College of Medicine, University of Arkansas for Medical Sciences, Little Rock, AR USA; 2 Department of Emergency Medicine, University of Arkansas for Medical Sciences, Little Rock, AR USA; 3 Fay W. Boozman College of Public Health, University of Arkansas for Medical Sciences, Little Rock, AR USA

**Keywords:** HVIP, PTSD, Mental health intervention

## Abstract

**Background:**

Violent crime is a prominent cause of injury in the United States and a significant public health issue. Hospital-Based Violence Intervention Programs (HVIPs) have emerged as multidisciplinary programs that improve wellbeing of violent assault victims and minimize recidivism through culturally competent bedside and post-discharge mentorship, connection to mental health and social services, and follow-up care. Because victims are prone to develop post-traumatic stress disorder (PTSD), it is important for HVIPs to engage in early PTSD screening and mental health interventions. Current research describing HVIP impacts on PTSD symptoms has not been aggregated; therefore, a systematic review was performed answering to what extent HVIP participation affects PTSD symptoms of violent assault victims.

**Methods:**

A literature search was performed on five databases on 07/21/2025. Inclusion criteria were studies that used valid measurement tools to report PTSD symptoms over time of U.S. violent assault victims who participated in an HVIP. Exclusion criteria were studies without hospital-based or hospital-linked violence intervention programs; studies including victims of violence other than interpersonal assault; and non-primary, non-peer-reviewed, or non-English sources. Articles were screened by title and abstract, then by full text using Rayyan software. Eligible articles were appraised using the Strengthening the Reporting of Observational studies in Epidemiology checklist.

**Results:**

616 non-duplicated articles were identified, of which four were included for final analysis. Two studies were single arm field trials, one was a quasi-experiment, and one was a pilot randomized controlled trial. Studies showed inconsistent effectiveness of HVIPs on PTSD symptoms, though two studies that tested a specific mental health intervention incorporated into an HVIP found significant symptom reduction. Sample characteristics, intervention components, and methodology were heterogenous across studies, impacting result comparability.

**Conclusion:**

While these studies show promise for PTSD symptom reduction, this review warrants more research to further elucidate the association between HVIP interventions and PTSD symptoms to better inform HVIP practices. Only 4 studies met inclusion criteria, indicating a lack of research evaluating this association, especially studies not testing an added specific mental health intervention. Moreover, findings support consideration for adding targeted mental health treatments within HVIPs to increase impact on PTSD symptoms.

## Introduction

In 2023, there were an estimated 2.2 million violent, nonfatal injuries in the United States (U.S.), over 70% of which would be classified as assault [1]. Over time, violent crime has come to be viewed as an issue of public health. Violent, nonfatal injuries are most common amongst young, poor, uneducated males of color, pointing to social determinants of health as a correlate to risk of violent injury [2]. Importantly, those who experience violent injuries are at risk of recidivism or retaliation, which increases risk of reinjury [3]. Recidivists who present to emergency departments on multiple occasions are at a higher risk of death with each subsequent visit and become more expensive to treat [3, 4].

According to the Health Alliance for Violence Intervention (HAVI), the national oversight body for Hospital-Based Violence Intervention Programs (HVIPs), HVIPs have arisen as a model to reduce risk of violent reinjury [5]. HAVI describes HVIPs as multidisciplinary programs that begin with violence prevention professionals engaging in bedside interventions at teachable moments while the victim recovers from injury. Importantly, violence prevention professionals are culturally competent social workers who often have the same background or lived experiences as the victims, allowing for a unique opportunity to build rapport and provide mentorship. Mentorship continues throughout the length of recovery and often many months after discharge. During and after recovery, victims are also connected with hospital- and community-based social services which might include mental health support, legal aid, job services, substance abuse treatment, educational training, and housing/relocation services [6]. These services, provided under the umbrella of case management, help address social determinants of health that perpetuate violent crimes in communities and overcome barriers to receiving care that many with poor socioeconomic status experience [5, 7].

As more HVIPs emerge across the U.S., research is needed to confirm that the standard HVIP model is effective at meeting all stakeholder needs. Systematic and scoping reviews of HVIPs have demonstrated that recidivism reduction is commonly used by hospital systems as a metric for program success [8–10]. This metric is practical because lowering recidivism risk decreases hospital costs, justifying program continuation [11, 12]. It is also important to consider if victim needs are met. One of the most frequently reported needs by HVIP participants is mental health services [6].

It is well-documented that victims of violent trauma are prone to experience mental health disorders including post-traumatic stress disorder (PTSD), depression, and substance use disorder [8–10]. PTSD is a mental condition that may arise after a person experiences a traumatic event. According to the Diagnostic and Statistical Manual of Mental Disorders, 5th Edition (DSM-5), a traumatic event as it relates to PTSD is defined as “exposure to actual or threatened death, serious injury, or sexual violence” [13]. The DSM-5 states that PTSD diagnostic criteria includes (A) the presence of intrusion symptoms such as distressing memories or dreams, dissociative reactions, or intense psychological distress; (B) avoidance of stimuli associated with the event; (C) negative alterations in cognition or mood following the event; (D) alterations in arousal or reactivity following the event; (E) duration of the disturbance must be greater than one month; and (F) the disturbance cannot be attributable to other causes such as medical conditions, medications, or substances. PTSD must cause impairment in social, occupational, or other areas and thus by definition is debilitating [13]. PTSD has been associated with functional impairment in general tasks of daily living as well as psychosocial impairment in relationships, community, and civic life [14].

Studies show that PTSD is correlated with a risk of re-offense [15]. Early interventions have been shown to reduce PTSD symptoms, so identifying individuals at risk of PTSD early in the recovery process can be beneficial [16]; therefore, it is important for HVIPs to engage in early PTSD screening and mental health interventions to both minimize recidivism and improve mental health. HVIPs should measure PTSD symptoms before, during, and after program completion to measure impact and ensure effectiveness in detecting and improving these symptoms. Success in mental health metrics could slow the cycle of violence in underprivileged communities.

Current research on the impacts of HVIPs on PTSD symptoms has not been aggregated. Therefore, a systematic review of current research was performed to address the following: to what extent does participation in HVIPs affect post-traumatic stress disorder (PTSD) symptoms of violent assault victims?

## Methods

### Criteria

Inclusion criteria were as follows:

1. Studies must analyze PTSD symptoms of violent assault victims at two or more times over the course of the study, including before, during, and after intervention.
2. Studies must measure PTSD symptoms of violent assault victims in the context of hospital-based or hospital-linked violence intervention programs.
3. Programs in the studies must be in the U.S. and be labeled as an HVIP by HAVI or otherwise contain standard HVIP criteria including (a) intervention at bedside or soon after discharge, (b) case management or linkage to services, (c) peer support, and (d) follow up care [17].
4. Studies must measure PTSD using validated measurements such as the PTSD Checklist for Diagnostic and Statistical Manual of Mental Disorder, 5th edition (PCL-5), PTSD Checklist-Civilian Version (PCL-C), or the Child PTSD Symptom Scale self-report (CPSS-SR-5) for DSM-5 [18–20].

Exclusion criteria were as follows:

1. Studies where no component of the program occurred in the hospital setting.
2. Studies that did not measure PTSD symptoms.
3. Studies with majority sexual assault, self-inflicted injury, disaster, war, terrorism, or domestic violence victims, as these populations generally fall beyond the intended scope of HVIPs.
4. Systematic reviews, case reports, meta-analyses, reviews of current literature, gray literature and non-peer-reviewed articles, and non-English articles.

No articles were excluded based on publishing date.

### Outcomes

The outcome of interest was PTSD scores. Several screeners have emerged as relevant for early detection of PTSD symptoms.

#### PCL-5

One of the most used measures for PTSD is the PCL-5, a 20-item self-report measure to screen individuals for PTSD, make a PTSD diagnosis, and monitor symptom change during and after treatment [19]. Questions are answered on a Likert scale ranging from 0 (not at all) to 4 (extremely). Studies have found it to be acceptably accurate in detecting PTSD amongst trauma-exposed individuals, including Black individuals [21, 22].

#### PCL-C

Weathers et al. states that in the 4th edition of the Diagnostic and Statistical Manual of Mental Disorders (DSM-4), an earlier version of the PTSD checklist was promoted. This version included the PCL-Specific (PCL-S), PCL-Military (PCL-M), and PCL-C [19]. One study found substantial agreement between the PCL-C, the most common of the three DSM-4 PTSD checklists, and the PCL-5 among military personnel and acutely injured trauma patients [23, 24]. The PCL-C checklist is a 17-item self-report measure with scores for each question ranging on a Likert scale from 1 (not at all) to 5 (extremely) [18].

#### CPSS-SR-5

An alternative checklist may be used for children ages eight to 18 called the CPSS-5, which is a 27-item checklist. 20 questions assess PTSD symptoms with scores for each question ranging on a Likert scale from 0 (not at all) to 4 (6 or more times a week/almost always), and the remaining 7 questions are yes/no questions to address impairment of daily functioning [20]. Studies have described that the CPSS has discriminant validity in measuring PTSD [25, 26].

### Search methods

Searches with specific keyword criteria as outlined in Appendix A were performed. To develop the query, terms were developed to return violence intervention programs, violent trauma with penetrating wounds with exclusion of domestic or self-inflicted violence, and PTSD. The search was performed on 07/21/2025 on PubMed, ProQuest, EBSCO, Embase, and Web of Science databases. The query terms were peer reviewed prior to database retrieval. The results were uploaded into Rayyan software for screening, and duplicates were removed.

### Data collection and analysis

Articles were screened first by title and abstract; for any article deemed eligible or possibly eligible upon title and abstract screening, a full-text screen was performed to confirm inclusion. All articles were screened independently by the lead author. Sources from all relevant systematic reviews in the initial search query and selected eligible articles were examined, with any relevant sources added to the list of studies as these articles were identified. A separate Microsoft Excel file with all articles and screening decisions was kept by the primary reviewer to maintain data integrity.

Selected eligible articles were appraised for completeness and transparency using the Strengthening the Reporting of Observational studies in Epidemiology (STROBE) guidelines [27]. The STROBE appraisal was completed by three authors to minimize errors and enable thorough review of each article. Data from these articles was extracted to the Microsoft Excel spreadsheet. Extracted information was adapted from Cochrane’s checklist of items to consider for data collection [28]. The data included:

1. Article title, author(s), year of publication, and Journal.
2. Sample Characteristics – sample size and participant demographics, type of injury.
3. Intervention components – intervention components, duration and frequency, setting, fidelity.
4. Study Design and methodology – study type, PTSD measurement tool, method of aggregation and analysis, bias and limitations.
5. Outcome – addressing incomplete data, summary data, secondary outcomes, conclusions.

Any data not available in the article were left blank in the Excel document and, if relevant, omission was noted.

## Results

**Fig. 1:**
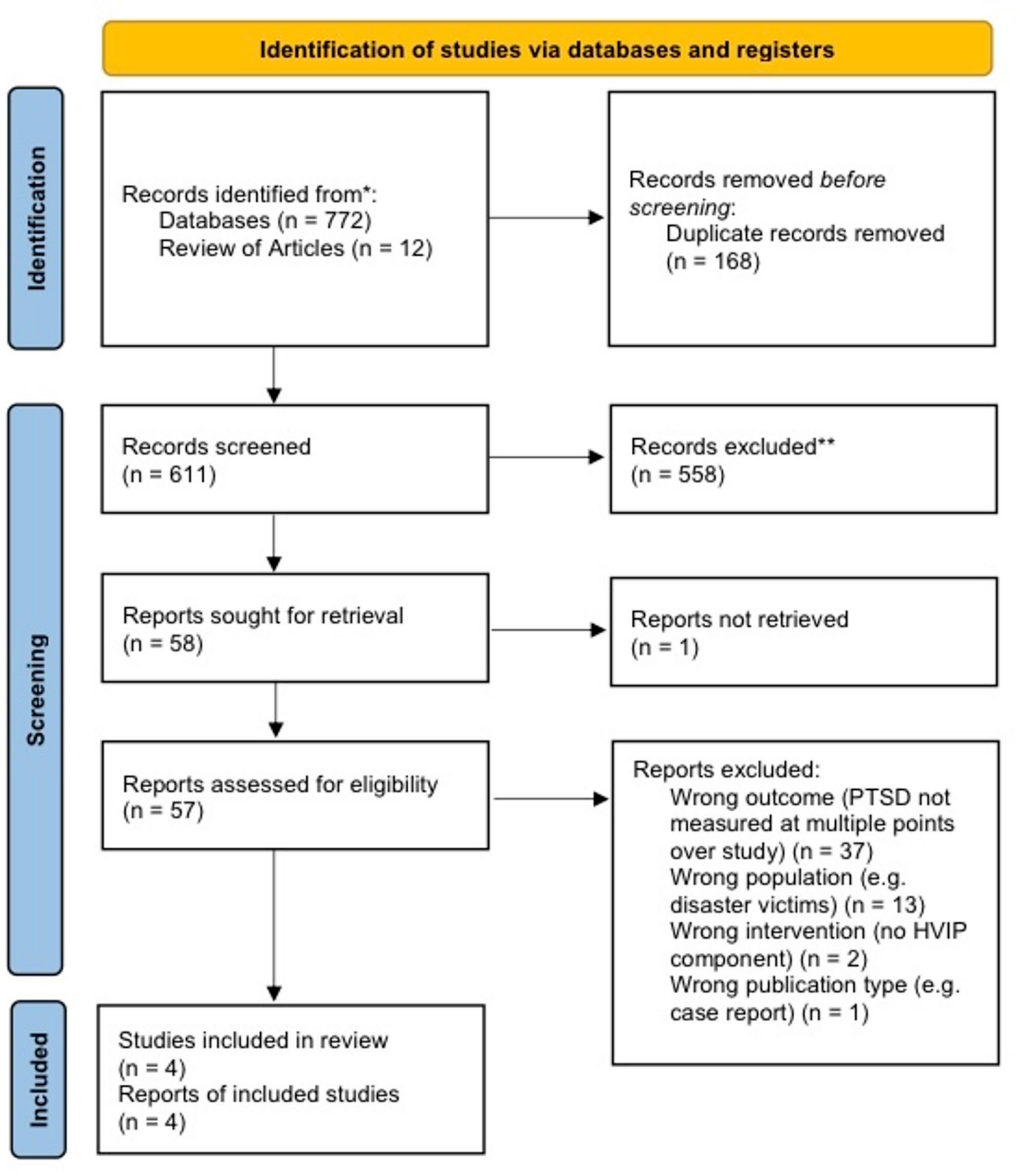
PRISMA flow diagram of screening and identification process

784 articles were identified from the five databases. 168 of these were duplicates and were removed. Of the 616 articles screened by title and abstract, 558 were excluded, leaving 58 articles for retrieval. 57 articles were successfully retrieved. After a full-text screening and based on inclusion/exclusion criteria, four articles were ultimately included for analysis. A PRISMA flowchart depicting the study identification and selection process is available in Fig. 1 [29].

A summary of key results for each included study is depicted in Table 1.

**Table 1:** Summary of key results for included studies

Article	Sample Characteristics			Intervention Components		Study Design and Methodology		Conclusions
	Sample Size	Age Range	Injury Type	Mental Health Intervention	Frequency and Time Period	Study Type	PTSD Measurement Tool	
Williams et al. [30]	100	13+	Non-self-inflicted GSW	SPR	45–60 min weekly for 5–6 weeks 2019–2022	Single arm field trial	PTSD Risk Screener + PCL-5	Receiving SPR in the early aftermath of a GSW significantly reduces PTSD.
Gorman et al. [12]	69	18–60	Intentional penetrating physical assault, GSW, or stab wound	HVIP referral services only	Variable 2017–2020	Quasi-experiment	PCL-5	HVIP participants had significantly less PTSD at hospital discharge, but no difference at 3 or 6 months.
O’Neill et al. [31]	14	18+	GSW	START	30–45 min one time 2020–2022	Pilot RCT	PCL-C	START decreased PTSD symptoms, but reduction was not significant.
Zinny et al. [32]	50	8–18	Physical assault, GSW, stab wound, or witnessed gun violence	TF-CBT	60–90 min for 16 sessions over 8 months, on average 2018–2020	Single arm field trial	CPSS-SR-5	TF-CBT completion resulted in significant improvement in PTSD symptoms.

GSW, gunshot wound; SPR, Skills for Psychological Recovery; PTSD, posttraumatic stress disorder; HVIP, hospital-based violence intervention program; PCL-5, PTSD Checklist for Diagnostic and Statistical Manual of Mental Disorder, 5th edition; START, Screening and Tool for Awareness and Relief of Trauma; RCT, randomized controlled trial; PCL-C, PTSD Checklist—Civilian Version; TF-CBT, trauma-focused cognitive behavioral therapy; CPSS-SR-5, Child PTSD Symptom Scale for DSM-5

### Sample characteristics

#### Sample size and participant demographics

All studies had sample sizes of 100 participants or less. Articles differed in age requirements for participation, with Zinny et al. exclusively including children and adolescents (mean age = 14) and Williams et al. allowing participants 13 and older (mean age 33) [30, 32]. Of note, in Gorman et al. the eligible age range changed over time due to funding restrictions, though all participants accepted were adults (median age = 29) [12].

In all studies, 50% or more participants identified as Black,. O’Neill et al. had the highest percentage of Black participants at 92.8% [31]. Notably, Zinny et al. the highest percentage of Latino participants at 46% [32]. Furthermore, a majority of participants in all studies were male [12, 30–32]. These demographics align with literature showing disproportionate rates of firearm violence amongst Black males [33].

Gorman et al. and O’Neill et al. required patients to speak English due to intervention language limitations, while Zinny et al. offered trauma-focused cognitive behavioral therapy (TF-CBT) in Spanish [12, 31, 32]. Language eligibility requirements are important because Latinos are also disproportionately affected by gun violence [34].

#### Type of injury

The most common injuries served in all studies were gunshot wounds (GSWs). Williams et al. and O’Neill et al. only accepted participants with GSWs [30, 31]. Gorman et al. and Zinny et al. expanded eligibility to other wounds, such as stab wounds [12, 32]. Zinny et al. included seven participants (14%) who were direct witnesses to violent injury; though this population is not typically served by HVIPs, these participants were in the minority, and other eligibility criteria were met, so the study was included [32]. In Gorman et al., similar to eligibility by age, injury eligibility changed over time due to funding limitations [12].

### Intervention components

#### Intervention components

All studies operated as part of an HVIP and commonly provided bedside interventions, mental health services, and case managers to support participants with social services such as legal appointments, medical appointments, transportation, housing, and job searches. Williams et al., O’Neill et al. and Zinny et al. tested specific mental health interventions designed to address trauma and build coping skills [30–32]. Specific interventions all sought to improve PTSD symptoms in violent assault victims and thus might be beneficial to integrate into HVIP mental health services.

Skills for Psychological Recovery (SPR) uses cognitive behavioral therapy (CBT) to facilitate recovery [30]. SPR is designed to be given as a follow-up to Psychological First Aid (PFA), which was provided at or soon after the initial encounter. Similarly, TF-CBT integrates trauma interventions with CBT to gradually expose details of the traumatic event and build skills and communication [32]. The entire program involved a sequential delivery of services that included outreach and engagement, active TF-CBT, and discharge planning and aftercare phases. Because Zinny et al. served exclusively adolescents, case manager duties were expanded to include attending school meetings, and program interventions occurred at home and school to remove barriers to receiving care [32]. Screening and Tool for Awareness and Relief of Trauma (START) helps develop techniques, including relaxation exercises, positive reframing, and structured problem-solving, to deal with posttraumatic stress [31]. No specific mental health intervention is specified in Gorman et al.; patients were referred to mental health services, which were provided in-house once funding allowed for hiring of a mental health provider [12].

O’Neill et al. offered START and Zinny et al. offered TF-CBT exclusively in-person [31, 32]. Williams et al. provided SPR in-person, via telehealth, or a combination of both [30]. It is important to note that follow-ups, including contact for surveys and case management, are commonly made over the phone due to participant and worker comfort with this mode of communication.

#### Duration and frequency

For Williams et al. O’Neill et al., and Zinny et al., which provided therapy over multiple time points, session timing was variable based on discretion of staff and availability of participants, and descriptions listed in Table 1 are averages [30–32]. In Gorman et al., case managers met with patients daily while inpatient, 1 to 2 times weekly for 2 to 4 weeks after discharge, and at 3-month intervals for 1 year [12]. Other services were provided at the discretion of the case manager, and frequency of provision of these services was highly variable.

#### Setting

Williams et al. and Gorman et al. were provided in a level I trauma center; Williams et al. was in the midwestern United States, while Gorman et al. was in New Jersey [12, 30]. Zinny et al. operated with an HVIP in Philadelphia that was linked to a local emergency department and local health, social services, and educational organizations, though the intervention took place in community, school, hospital, and office settings [32]. O’Neill et al. was set in New Haven, CT [31].

Gorman et al. and O’Neill et al. only allowed enrollments from the hospital that contained the HVIP program [12, 31]. Williams et al. and Zinny et al. accepted both patients from the hospital containing the HVIP program and referrals from external and community partners [30, 32]. Zinny et al. specified that referrals came from local health, social services, and education organizations [32].

All studies were in some way impacted by the COVID-19 pandemic, forcing the programs to shut down or suspend certain services. During this time, therapy was only available via telehealth, with all surveys conducted over the phone. Williams et al. notes the impact of COVID-19 on their study is unclear—on one hand, it may have undermined the effectiveness of recruitment and intervention strategies; on the other hand, worsened mental health during the time may have encouraged more individuals to seek mental health services [30].

#### Fidelity

O’Neill et al. provides the most thorough assessment of intervention fidelity [31]. The pilot study measured a significant difference in time spent with treatment and control groups due to time spent performing the START intervention. Sessions were recorded and the authors reported survey and audio recordings were in accordance 81.3% of the time, with one recording missing because of equipment failure. 81.3% fidelity is quite low, which signaled to the authors that a simple survey is most beneficial for those administering the intervention to ensure critical intervention components are completed.

Gorman et al. made no mention of fidelity checks, and Williams et al. stated that SPR sessions were not coded for fidelity but were discussed during weekly group supervision to promote protocol adherence [12, 30]. Zinny et al. stated that a fidelity check for TF-CBT, case management, and peer support was performed by the lead author for over 300 case notes through a TF-CBT Brief Fidelity Checklist, with confirmation that all components were provided with fidelity; no quantitative value was provided for compliance [32].

### Study design and methodology

#### Study design

In reference to the information presented in Table 1, three studies tested a specific mental health intervention as part of an HVIP, so the contribution of general HVIP services to PTSD symptom reduction is unclear.

#### Recruitment procedures

Two studies offered monetary incentives at certain points over a study. In O’Neill et al., beginning in September of 2020, a $40 cash gift card was provided as an incentive to join the study and recruitment expanded to the trauma clinic to bolster enrollment [31]. Gorman et al. offered $25 on a reloadable Clincard for completion of time point 2 and time point 3 surveys [12].

#### Measurement tool

All articles used measurement tools outlined in the inclusion criteria. Williams et al. used a 5-item PTSD Risk Screener on the initial encounter, adding to the heterogeneity of outcomes [30].

#### Method of aggregation and analysis

The statistical methods used to analyze interventions and their impact on PTSD symptoms differed widely across articles. Both Gorman et al. and O’Neill et al. used Fisher’s exact test, but Gorman et al. combined this with logistic regression and repeated measures ANOVA, while O’Neill et al. combined this with the Kruskal-Wallis test and Wilcoxon signed rank test [12, 31]. Zinny et al. also used a Wilcoxon signed rank test in addition to an unpaired t-test and Cohen’s d [32]. Williams et al. used bivariate analyses and a mixed effects model [30]. Similar to the measurement tools, the heterogenous analyses limits the ability to draw interpretations across all studies and precludes meaningful aggregation.

Furthermore, studies expressed different cadences of outcome measurements. Beyond baseline surveys, Gorman et al. completed additional surveys at 3 and 6 months after hospital discharge [12]. Zinny et al. only had an additional survey time point after TF-CBT completion, which on average took 9 months [32]. O’Neill et al. had additional surveys that were completed within 6 weeks and between 3- and 6-months post-injury [31]. Williams et al. administered surveys weekly after each SPR session [30].

#### Bias and limitations

As previously mentioned, all studies overlapped to some extent with the COVID-19 pandemic, which studies noted may not be generalizable to different time periods. Furthermore, generalizability may be limited by use of convenience sampling, though all studies had majority Black and Latino males, populations most impacted by gun violence [34]. All studies also cited low sample size as a limitation, which leads to a cautious interpretation of results. This can be compounded by low response rates for surveys, which might occur if participants are reluctant to disclose information or trust providers or the medical system [12].

Given the lack of fidelity checks in several articles, heterogeneity in service delivery is a limitation. For example, Gorman et al. commented that an in-house mental health provider was hired once funding was obtained, allowing patients easier access to mental health services [12]. This increased accessibility demonstrates a move to better patient care, but it could prove true that those who completed in-house therapy had less barriers to care or were more motivated to get better, which represents a potential source of bias.

O’Neill et al. and Zinny et al. had no control group, which further limits the ability to draw causal inferences from the results [31, 32]. Finally, Williams et al. mentioned that using PFA as a precursor to SPR limits the determination of any PTSD impact from SPR, however, this is difficult to separate as SPR is intended to be a follow-up therapy to PFA [30].

### Outcomes

#### Summary data

Single arm field trials showed significant PTSD symptom reduction after intervention [30, 32]. Zinny et al. showed that youth who completed TF-CBT showed a 49.5% decrease in PTSD post-test [32]. In their mixed model analyses, Williams et al. found a 4.32-point decrease in PCL-5 scores for each session of SPR [30]. For Gorman et al., the quasi-experiment, PTSD symptoms of HVIP participants were only significantly less than non-HVIP participants at time of discharge; 3- and 6-months showed no significant difference dependent on HVIP participation [12]. In the pilot randomized control trial (RCT) of O’Neill et al., which had low statistical power, the control group saw a 0.4-point decrease from baseline to 1 month and a 5.8-point decrease from 1 to 3–6 months in PCL-C scores, while the treatment group saw a 10.7-point decrease from baseline to 1 month and 3.4-point increase from 1 to 3–6 months; PTSD symptom reduction for the intervention group may prove significant if held over a larger sample [31].

#### Secondary outcomes

Secondary outcomes are relevant because PTSD often coexists with other mental health issues [35]. O’Neill et al. measured insomnia using the Insomnia Severity Index (ISI) before and after the START intervention but found no significant difference [31]. Williams et al. and Zinny et al. both measured depression with mixed results [30, 32]. Using mixed effects model, Williams et al. found a 1.55-point decrease in the Patient-Reported Outcomes Measurement Information System (PROMIS) depression scores for each session of SPR [30]. Zinny et al. found no statistical difference in depression scores at baseline between completers and non-completers of TF-CBT using the Short Mood and Feelings Questionnaire (SMFQ) [32].

In comparing telehealth to in-person therapy, Williams et al. found that telehealth participants were more likely to complete 3 or more SPR sessions and had statistically similar outcomes to in-person participants [30]. Thus, modality of delivery can program success in different ways, including effectiveness of the therapy and accessibility for participants.

The study with Gorman et al. had secondary aims that included determining whether HVIP participants met stated goals and qualitative interviews to gauge effectiveness of HVIP [12]. Authors found that 49% (*n* = 146) of HVIP participants were able to achieve their stated goals; achievement was determined at the discretion of the case managers. Prominent themes from qualitative interviews included relationships with case managers, the success of the HVIP model, revenge and retaliation, and safety and protection.

#### Addressing incomplete data

As previously mentioned in bias and limitations, risk of attrition bias was high for these studies except for O’Neill et al. because the intervention was completed in a singular session [12, 30–32]. Zinny et al. had the highest full-program completion percentage (*n* = 29, 58%) and Gorman et al. had the lowest (*n* = 14, 20%) [12, 32]. One theory is that COVID-19 may have impacted attrition due to added social and financial stress as well as challenges in technology utilization, though this would need to be studied. Incentives for participation were provided in O’Neill et al. and Gorman et al., which is one method to improve recruitment and retention [12, 31]. Besides notation in the manuscript, no studies resolved missing data, though Williams et al. used a mixed methods model that can address missing data [30].

#### Conclusions

Results from the four studies were mixed, but commonalities included that targeted mental health interventions resulted in more positive outcomes compared to general mental health referral services [12, 30–32]. As is the mission of HVIPs, early intervention is commonly emphasized as vital to recruitment and success [12, 30, 31]. Zinny et al. emphasized providing “holistic, culturally relevant services” to HVIP participants [32]. Williams et al. concluded that modality will not moderate outcomes [30]. found that the proposed RCT for the START intervention is feasible, and they recommend modifications such as development of audiovisual resources for convenience [31]. Finally, Gorman et al. concluded that early effectiveness for an HVIP intervention is best measured in meeting immediate health and social needs of violently injured patients; additionally, PTSD maybe a confounding factor in HVIP success, and HVIPs may have an opportunity to address PTSD symptoms [12].

## Discussion

Given that only four studies met inclusion criteria, there is a lack of research evaluating the impact of HVIPs on PTSD symptoms in violent assault victims, especially studies not testing an added specific mental health component. The available data from these studies indicate more targeted mental health interventions like SPR and TF-CBT show promise in treating PTSD symptoms compared to general HVIP referral services, perhaps suggesting more novel therapy methods should be incorporated into the HVIP model [12, 30, 32]. Gorman et al. and O’Neill et al. showed no difference in PTSD symptoms for intervention versus control groups except at hospital discharge in the Gorman et al. study [12, 31]. Therefore, it is difficult to say if these interventions or some other factor is contributing to PTSD decline. More evidence, especially from RCTs, will be needed to elucidate the association between HVIP interventions and PTSD symptoms to better inform future HVIP practices. Several articles were found describing protocols for HVIPs and include collection of PTSD data, which is promising for future data on the subject [36, 37].

Based on this review, one aspiration for future studies such as those listed above is the inclusion of larger sample sizes for increased statistical power. Power is a concern for the four included studies due to small sample sizes, which could lead to false negative results and a lack of data reproducibility [12, 30–32]. Larger studies may not be feasible given HVIP complexity and expensiveness, but additional data points would reduce uncertainty.

Uncertainty is also introduced in the included studies by incomplete or insufficient fidelity checks. Given that HVIPs are complex and require teamwork across roles such as social workers, trauma department and emergency department staff, case managers, peer specialists, community partners, mental health experts, and others, fidelity checks are important to improve patient care and protect data integrity. Only O’Neill et al. and Zinny et al. performed fidelity checks, meaning Gorman et al. and Williams et al. lacked any method of ensuring standardization of procedures across HVIP and early intervention mental health therapy [12, 30–32]. Effort is needed in future studies to confirm interventions are implemented with fidelity and ensure the HVIP meets quality of care standards, acts ethically, and maintains data integrity. Without fidelity checks, the intervention may not be homogenous for participants, decreasing internal validity and generalizability of the study.

Generalizability and reproducibility are also impacted by the time period of operation, as all studies overlapped with the COVID-19 pandemic, which impacted intervention implementation; studies had to suspend operations and/or provide virtual services [12, 30–32]. COVID-19 caused an increase in psychological distress across the world [38]. As Williams et al. states, there is a lack of clarity in how the COVID-19 pandemic might influence study results [30]. Furthermore, retention might have been impacted if participants found using online forms of communication burdensome [39]. Thus, the COVID-19 pandemic uniquely impacted institutions and participants, including in the transition of service delivery to incorporate virtual formats.

Studies have supported the finding from Williams et al. that virtual and in-person therapy are equally effective at treating PTSD; however, it has yet to be tested if other aspects of HVIPs are effective at treating PTSD symptoms or other mental health disorders if delivered in a virtual format [30, 40]. Though virtual services may increase accessibility, contacting HVIP patients virtually may prove difficult because of limited access to technology or unreliable service and unfamiliarity with online applications, leading to a potential self-selection bias; furthermore, those with mental health issues may find completely virtual services isolating [41, 42].

All included studies were subject to potential sources of bias, including sampling bias, measurement bias, and attrition bias, which may impact internal and external validity [12, 30–32]. Articles may experience measurement bias with the PCL-C, PCL-5, and CPSS-SR-5 tools. Though these PTSD measurement tools are widely accepted as reliable and valid measures of PTSD symptoms, they are not homogenous, so synthesizing or performing a meta-analysis of the results is not possible. This limits the interpretations that can be drawn across the studies, and it must be accepted that some result differences could be due to a difference in measurements.

Interpretations may also be limited by sampling bias. The studies used convenience sampling methods, as those eligible for participation in the HVIP or mental health treatment either presented to the hospital or were referred; however, HVIP samples will likely continue to be nonrandom because the participant population is limited to those who present to the institution or community partners with a violent assault injury. Since certain demographics such as Black males and those from underprivileged communities are most burdened by gun violence, study samples will be heavily skewed towards these populations, as was evident in the included studies, which limits generalizability to other populations [12, 30–32, 34].

HVIPs are also typically skewed in terms of injury type, and this was true of the included studies. Exclusion of injury types is purposeful because HVIPs traditionally serve victims of intentional, non-self-inflicted assault, which largely contribute to the cycle of violence and retaliation that perpetuates underprivileged communities. In tailoring support and treatment for individuals who meet criteria, HVIPs can better achieve their mission of reducing reinjury through interpersonal violence [5].

The HVIPs studied were additionally skewed in terms of geographical location. The included studies were confined to the Midwest and Northeastern United States, which are the 2 regions with the most HVIPs, but data from different regions could provide a more complete picture of how HVIPs impact participants and elucidate regional nuances that facilitate HVIP success [12, 30–32]. For example, an HVIP in Texas or Arizona would be better positioned to serve a large demographic of Latinos and thus should highly consider offering Spanish translational services. Accessibility to public transit could also impact the preferred modality of therapy services. Expanding the geographical scope of HVIP analysis will be important as programs are established throughout the United States.

Another important demographical consideration for HVIPs is eligibility of adolescents. If an HVIP chooses to treat adolescents, protocols and services should be tailored to fit needs of the population, which includes different evaluations and mental health therapies more appropriate for children in mental distress, involvement of a parent or caregiver in consent and various services, accompaniment to school meetings, and developmentally appropriate communication and education [43]. Zinny et al. successfully incorporated many of these services into the program, demonstrating feasibility in pediatric populations [32]. As adolescents who engage in violent crime are likely to be involved in repeat incidents, HVIPs that can successfully reach this population may be well positioned to interrupt the cycle of violence before recidivism becomes chronic [44].

Hospital-based programs versus hospital-linked programs may face different challenges in terms of recruitment and retention of participants, but there is a significant gap in research on this topic. HVIPs that accept referrals from community organizations may have a broader scope, serving larger geographical areas and harder-to-reach communities compared to strictly hospital-based HVIPs. It may prove true that HVIPs that accept community referrals exhibit stronger community partnerships and are thus able to better serve participants. On the other hand, hospital-based HVIPs may have more control over early bedside intervention, which may improve enrollment and retention due to patient access at teachable moments, a concept emphasized by HVIPs [5]. These are all conjectures that need to be tested. Two programs included in this study also offered incentives to enhance enrollment and retention, which was commonly listed as a study limitation [12, 31, 32].

Regardless of the HVIP structure, flexibility in providing services to participants is a theme that emerged from all studies. Studies stressed the need to increase accessibility for victims of violent crimes to address individual circumstances, where possible, which might include offering Spanish translation or audiovisual resources for impaired individuals [31, 32]. Accessibility may also prove relevant for the frequency and duration of mental health therapy, which differed in the included studies. Research has shown increased frequency of PTSD therapy improves outcomes, but professionals must balance this knowledge with a realistic perspective of the availability and motivation of participants to maximize therapy benefits while minimizing time burdens [45].

### Limitations

There are several limitations to this systematic review. First, included studies varied significantly in study design, interventions, and measure tools. The studies included were two single arm field trials, one quasi-experiment, and one pilot RCT. Only one study purely tested the impact of an HVIP in its entirety on PTSD symptoms. This variability means synthesis and comparison of results on the topic is a challenge. Further research is required to more thoroughly address the research questions. Second, only one author performed the article screening, which could lead to bias in article inclusion or exclusion. Having at least one additional screener would have provided a more robust screening process. Third, the keyword search performed for this study was too sensitive, as it pulled many articles on PTSD in disaster, war, and terrorism victims, all of which are generally outside of the scope of an HVIP target population. The exclusion criteria were modified so that these studies could be properly excluded, however, curating a more specific search strategy would have been more effective and possibly introduce less bias and errors in screening. The exclusion criteria were also modified so that studies with a minority of participants who were victims of sexual assault, self-inflicted injury, or domestic abuse could be included, as many HVIPs still accepted some participants with these injury types. Finally, one article for which full text was requested was unable to be located, which could mean eligible articles were missed in the screening process.

## Conclusion

This systematic review addressed to what extent participation in HVIPs affect PTSD symptoms of violent assault victims. Results were inconclusive as to the impact of HVIPs, though targeted early mental health interventions may be more impactful in reducing PTSD symptoms. For HVIPs around the U.S., this study highlights the importance of collecting robust mental health data and striving to accommodate individual patient needs, especially accessibility and flexibility in delivering services. Additionally, programs may consider implementation of specialized early intervention mental health treatment to more appropriately address violent assault victims’ mental health needs. As more HVIPs become established and research grows, a more thorough systematic review on this topic is recommended. This research could also be extended to other mental health disorders such as depression and other anxiety disorders, which commonly coexist with PTSD [35].

## Data Availability

The datasets used and/or analyzed during the current study are available from the corresponding author on reasonable request. The search query used is available in Appendix 1.

## Appendix A: keyword search

(“Crisis Intervention“[Mesh] OR “HVIP” OR “violence intervention*” OR “hospital-based violence intervention*” OR “hospital based violence intervention*” OR “hospital violence intervention*” OR “hospital-based early intervention*” OR “hospital based early intervention*” OR “community violence intervention*” OR “community based violence intervention*” OR “community violence prevention*” OR “community based violence prevention*”) Sort by: Most Recent.

AND

(“Violence“[Mesh] OR “violent trauma*” OR “gunshot wound*” OR “gun” OR “guns*” OR firearm* OR “stab*” OR “assault*” OR “violen*” OR “interpersonal violence*” OR “deliberate injur*” OR “deliberate trauma*” OR “inflicted injur*” OR “inflicted trauma*” OR “intentional injur*” OR “nonaccidental injur*” OR “non-accidental injur*” OR “nonaccidental trauma*” OR “non-accidental trauma*” OR “violent injur*” OR “penetrating trauma*” OR “penetrating assault*” OR “penetrating injur*” OR “penetrating wound*”)

NOT

(domestic* OR “domestic violence*” OR “domestic assault*” OR sexual* OR “sexual violence*” OR “sexual assault*” OR “intimate partner*” OR “suicid*” OR “self-inflicted trauma*” OR “self inflicted trauma*” OR “self-inflicted wound*” OR “self inflicted wound*”) Sort by: Most Recent.

AND

(“Trauma and Stressor Related Disorders“[Mesh] OR PTSD* OR PTS OR “post traumatic stress*” OR “post-traumatic stress*” OR “posttraumatic stress*” OR “acute stress disorder*” OR “anxiety” OR “traumatic event*” OR CPTSD OR “complex posttraumatic stress*” OR “complex post traumatic stress*” OR “complex PTSD” OR “acute stress reaction*” OR “trauma reminder*” OR “avoidance*” OR “negative alterations in mood” OR “alterations in arousal”) Sort by: Most Recent.

## Abbreviations

CDC: Center for Disease Control and Prevention
HAVI: The Health Alliance for Violence Intervention
HVIP: Hospital-based violence intervention program
PTSD: Post-traumatic stress disorder
DSM-5: Diagnostic and Statistical Manual of Mental Disorders, 5th edition
PRISMA: Preferred Reporting Items for Systematic Reviews and Meta-Analyses
PCL-5: PTSD Checklist for DSM-5
PCL-C: PTSD Checklist-Civilian Version
CPSS-SR-5: Child PTSD Symptom Scale for DSM-5
DSM-4: Diagnostic and Statistical Manual of Mental Disorder, 4th edition
PCL-S: PTSD Checklist-Specific Version
PCL-M: PTSD Checklist-Military Version
STROBE: Strengthening the Reporting of Observational studies in Epidemiology
GSW: Gunshot wound
TF-CBT: Trauma-focused cognitive behavioral therapy
SPR: Skills for Psychological Recovery
CBT: Cognitive behavioral therapy
PFA: Psychological First Aid
START: Screening and Tool for Awareness and Relief of Trauma
RCT: Randomized controlled trial
ISI: Insomnia Severity Index
PROMIS: Patient-Reported Outcomes Measurement Information System
SMFQ: Short Mood and Feelings Questionnaire
